# Sirtuin 5 Deficiency Does Not Compromise Innate Immune Responses to Bacterial Infections

**DOI:** 10.3389/fimmu.2018.02675

**Published:** 2018-11-20

**Authors:** Tytti Heinonen, Eleonora Ciarlo, Charlotte Théroude, Aimilia Pelekanou, Jacobus Herderschee, Didier Le Roy, Thierry Roger

**Affiliations:** Infectious Diseases Service, Department of Medicine, Lausanne University Hospital, Epalinges, Switzerland

**Keywords:** sirtuin, innate immunity, cytokine, macrophage, endotoxemia, sepsis, histone deacetylase, metabolism

## Abstract

Sirtuin 5 (SIRT5) is a member of the family of NAD^+^-dependent lysine/histone deacetylases. SIRT5 resides mainly in the mitochondria where it catalyzes deacetylation, demalonylation, desuccinylation, and deglutarylation of lysine to regulate metabolic and oxidative stress response pathways. Pharmacologic inhibitors of SIRT5 are under development for oncologic conditions, but nothing is known about the impact of SIRT5 on antimicrobial innate immune defenses. Using SIRT5 knockout mice, we show that SIRT5 deficiency does not affect immune cell development, cytokine production and proliferation by macrophages and splenocytes exposed to microbial and immunological stimuli. Moreover, preclinical models suggest that SIRT5 deficiency does not worsen endotoxemia, *Klebsiella pneumoniae* and *Streptococcus pneumoniae* pneumonia, *Escherichia coli* peritonitis, listeriosis, and staphylococcal infection. Altogether, these data support the safety profile in terms of susceptibility to infections of SIRT5 inhibitors under development.

## Introduction

Innate immune cells such as monocytes/macrophages, neutrophils and dendritic cells (DCs) express pattern recognition receptors (PRRs) that mediate the sensing of microbial associated molecular patterns (such as endotoxin, lipoproteins, peptidoglycans, glucans, mannans, and nucleic acids) and danger-associated molecular patterns released by injured or infected cells. PRRs encompass Toll-like receptors (TLRs), C-type lectin receptors, NOD-like receptors, RIG-I-like receptors, and cytosolic DNA sensors (1, 2). Upon ligand sensing, PRRs initiate intracellular signaling cascades remodeling host transcriptome to promote cytokine/chemokine production and the development of antimicrobial effector mechanisms. Innate immune responses have to be tightly regulated to avoid imbalanced life-threatening immune responses.

Sirtuins belong to the highly conserved family of NAD^+^-dependent lysine/histone deacetylases (HDACs). The seven mammalian sirtuins (SIRT1-7) are characterized by their domain organization, enzymatic activity and subcellular nuclear, nucleolar, cytoplasmic or mitochondrial localization. Sirtuins catalyze enzymatic reactions beyond deacetylation, and can function as ADP-ribosyltranferase, demyristolase, decrotonylase, desuccinylase, deglutarylase, demalonylase, deformylase, and demyristolase (3–7). Proteome analyses identified thousands of targets of sirtuins, and sirtuins have been involved in the regulation of many biological functions and pathological processes. Sirtuins are promising therapeutic targets for metabolic, cardiovascular, neurodegenerative, and oncologic diseases (3–7).

SIRT5 is one of the least characterized sirtuins. SIRT5 belongs, together with SIRT3 and SIRT4, to the so-called mitochondrial sirtuins. SIRT5 also localizes into the cytoplasm (8). SIRT5 was initially shown to deacetylate carbamoyl phosphate synthase (CPS1) to promote urea cycle (9). SIRT5 is a weak deacetylase and recent data suggest that SIRT5 primarily performs lysine demalonylation, desuccinylation, and deglutarylation (10, 11). SIRT5 desuccinylates and deglutarylates CSP1 to increase ammonia detoxification and desuccinylates 3-hydroxy-3-methylglutaryl-CoA synthase 2 to increase ketogenesis (10–12). SIRT5 desuccinylates succinate dehydrogenase and pyruvate dehydrogenase to repress cellular respiration and activates superoxide dismutase 1 and isocitrate dehydrogenase 2 through desuccinylation and glucose-6-phosphate dehydrogenase through deglutarylation. In this way, SIRT5 regulates NADPH homeostasis, scavenges reactive oxygen species (ROS), and increases resistance to oxidative stress (8, 13, 14). A malonylome analysis in liver identified gluconeogenesis and glycolysis as the most enriched pathways regulated by SIRT5 (15), while succinylome analyses of heart and liver identified fatty acid oxidation (FAO), amino acid metabolism and TCA cycle (8, 12, 16). Overall, SIRT5 is emerging as a key regulator of metabolism. SIRT5 protects from cardiac dysfunctions and dextran sulfate sodium-induced colitis and promotes or restricts cancer growth depending of the context (16–18). Thus, SIRT5 is a potential therapeutic target for several pathological conditions. Efforts are currently devoted to the generation of SIRT5 inhibitors such as thiosuccinyl peptides, cyclic pentapeptide harboring a central N(ε)-carboxyethyl-thiocarbamoyl-lysine residue and 3-arylthiosuccinylated and 3-benzylthiosuccinylated peptide derivatives (19–21) for specific cancer types (18, 22).

The impact of SIRT5 on antimicrobial host defenses is unknown; which is an important missing piece considering the clinical development of SIRT5 inhibitors. Using SIRT5 knockout mice, we show that SIRT5 deficiency has no major impact on immune cell development and on the response of macrophages and splenocytes to microbial stimulation. Going well along with these observations, preclinical models revealed that SIRT5 knockout mice are not particularly sensitive to endotoxemia, *Klebsiella pneumoniae* and *Streptococcus pneumoniae* pneumonia, *Escherichia coli* peritonitis, listeriosis and staphylococcal infection. Up to now, these data support the assumption that SIRT5 inhibitors should not increase patients' susceptibility to infections.

## Materials and methods

### Ethics statement

Animal experimentation was approved by the *Service de la Consommation et des Affaires Vétérinaires* of *Canton de Vaud* (Epalinges, Switzerland) under authorizations n°VD 3287, 876.8, 876.9, 877.8, and 877.9 and performed according to Swiss and ARRIVE guidelines.

### Mice, cells and reagents

Experiments were performed using 8 to 12-week-old C57BL/6J mice (Charles River Laboratories, Saint-Germain-sur-l'Arbresle, France) and SIRT5 knockout mice (kindly provided by Prof Johan Auwerx, Ecole Polytechnique Fédérale de Lausanne, Lausanne, Switzerland) backcrossed 7 times on a C57BL/6J background (23). Mice were housed (12 h light/dark cycle, 22°C, 70% humidity) under specific pathogen-free conditions in the animal facility of the Centre des Laboratoires d'Epalinges (CLE, Epalinges, Switzerland, license number VD-H04). Colonies were free of mouse norovirus and mouse hepatitis virus infections. Mice were fed with γ-irradiated food (Global Rodent XP 18, Provimi Kliba AG, Kaiseraugst, Switzerland) and water *ad libitum*. Mice were transferred in a BSL2 unit to perform *in vivo* models of infection.

Bone marrow-derived macrophages (BMDMs) and splenocytes were obtained and cultured as described (24, 25). For experiments, cells were seeded in complete medium without growth factors and antibiotics (1 or 20 × 10^5^ cells in 96 or 6-well plates). Stimuli were *Salmonella minnesota* ultra pure LPS (InvivoGen, San Diego, CA), Pam_3_CSK_4_ (EMC microcollections, Tübingen, Germany), CpG ODN 1826 (CpG, InvivoGen), toxic shock syndrome toxin-1 (TSST-1, Toxin Technology, Sarasota, FL), concanavalin A (Sigma-Aldrich, St. Louis, MI), anti-CD3ε, and anti-CD28 antibodies (clones 145-2C11 and 37.51, eBioscience, San Diego, CA) and phorbol-12-myristate-13-acetate (PMA) plus ionomycin (Sigma-Aldrich) or bacteria. Clinical strains of *E. coli* O18, *S. aureus* AW7, *K. pneumoniae, S. pneumoniae*, and *L. monocytogenes* 10403s were grown in brain heart infusion broth (BD Biosciences, Erembodegem, Belgium), washed in 0.9% NaCl and adjusted at 10^9^-10^10^ CFU/ml (26–29). Bacteria were heat-inactivated for 2 h at 56°C for *in vitro* use.

### Flow cytometry analyses

Single cell suspensions from thymus and spleen were enumerated and incubated with 2.4G2 monoclonal antibody (mAb) (30). Cells were stained using mAbs listed in Table S1. Data were acquired using a LSR II flow cytometer (BD Biosciences) and analyzed using FlowJo Version 10.2 software (FlowJo LLC, Ashland OR) (31).

### Western blot analyses

Protein extracts were submitted to PAGE and transferred onto nitrocellulose membranes (32–34). Membranes were incubated with antibodies directed against SIRT5 (8782, 1:1,000, Cell Signaling Technology, Danvers, MA) or β-actin (4967S, 1:1,000, Cell Signaling Technology) and then with a secondary HRP-conjugated antibody (31460, 1:10,000, Thermo Fisher, Waltham, MA) (35). Blots were imaged with the ECL Western blotting system (GE Healthcare, Little Chalfont, UK). Images were recorded using a Fusion Fx system (Viber Lourmat, Collégien, France) (36).

### Metabolic activity measurements

The oxygen consumption rate (OCR, in pmole O_2_/minute) and the extracellular acidification rate (ECAR, in mpH/minute) were analyzed using a 96-well format Seahorse XFe® system, the Seahorse XF Cell Mito Stress Test Kit and the Seahorse XF Glycolysis Stress Test Kit (Agilent Technologies, Santa Clara, CA). Four × 10^4^ BMDMs were plated in 96-well plates in complete IMDM medium. The next day, cells were rested one hour in Seahorse medium with or without glucose. Mitochondrial respiration was assessed by measuring OCR following the addition of 1 μM oligomycin (OM), 1 μM FCCP and 2 μM antimycinA/1 μM rotenone (AA/Rot). Glycolytic function was assessed by measuring ECAR following the addition of 10 mM glucose, 1 μM oligomycin and 50 mM 2-deoxy-glucose (2-DG).

### RNA analyses

Total RNA was isolated, reverse transcribed (RNeasy and QuantiTect reverse transcription kits, Qiagen, Hilden, Germany) and used in real-time PCR using Fast SYBR® Green Master Mix and a QuantStudio™ 12K Flex system (Life Technologies, Carlsbad, CA) as reported (24, 37). Samples were tested in triplicate. Gene specific expression was normalized to actin expression. Primers are listed in Table S2. Sirt5 mRNA expression levels in organs were extracted from the BioGPS resource (http://biogps.org).

### Proliferation and cytokine measurements

The proliferation of splenocytes cultured for 48 h in 96-well plates was quantified by measuring ^3^H-thymidine incorporation over 18 h (38). Cytokine concentrations were quantified using DuoSet ELISA kits (R&D Systems, Abingdon, UK) (39). The viability, assessed using the MTT assay (40), of resting and stimulated SIRT5^+/+^ and SIRT5^−/−^ BMDMs was not different.

### *In vivo* models

Mice were challenged intraperitoneally (i.p.) with 20 mg/kg LPS or 4 × 10^2^ or 3 × 10^4^ CFU *E. coli* O18, intranasally (i.n.) with 30 CFU *K. pneumoniae* or 1 × 10^6^ CFU *S. pneumoniae* and intravenously (i.v.) with 1.2 × 10^3^ or 9 × 10^4^ CFU *L. monocytogenes* or 3 × 10^4^ or 2 × 10^7^ CFU *S. aureus*. Blood was collected to quantify cytokines and bacteria (24). At least once daily, body weight, severity score (graded from one to five) and survival were registered (41). Animals were euthanized when they met a severity score of four. Two operators performed animal follow-up.

### Statistical analyses

Comparisons between different groups were performed by analysis of variance followed by two-tailed unpaired Student's *t*-test. *In vivo* bacteria and cytokine data were analyzed using the Mann-Whitney test. Survival curves were built using the Kaplan-Meier method and differences were analyzed by the log-rank sum test. All analyses were performed using PRISM (GraphPad Software). *P* values were two-sided, and *P* < 0.05 was considered to indicate statistical significance.

## Results

### SIRT5 deficiency has no major impact on the development of immune cells

**Figure 1:**
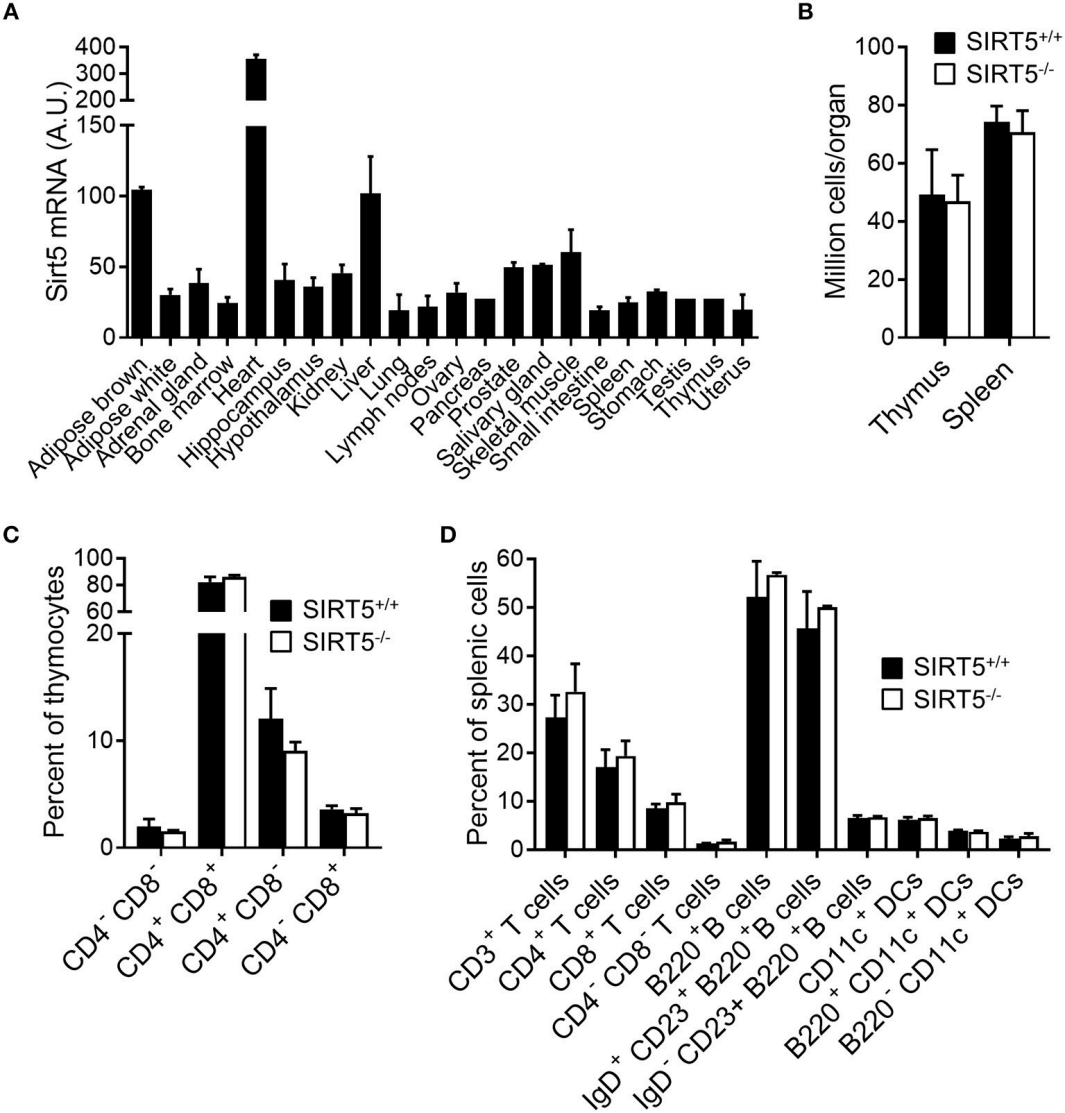
SIRT5 deficiency does not affect immune cell development in thymus and spleen. **(A)** Sirt5 mRNA expression levels in a panel of mouse organs (extracted from the BioGPS resource). **(B)** Total cell numbers per thymus and spleen of SIRT5^+/+^ and SIRT5^−/−^ mice. **(C,D)** Percentages of T cell subpopulations in the thymus **(C)** and of T cells, B cells and DCs subpopulations in the spleen **(D)** of SIRT5^+/+^ and SIRT5^−/−^ mice. Data are means ± SD from one experiment performed with three mice and are representative of two experiments. *P* > 0.5 for all conditions **(B–D)**.

SIRT5 mRNA was ubiquitously expressed in organs, including immune organs (bone marrow, lymph nodes, spleen, and thymus). Highest levels were observed in brown adipose tissue, heart and liver (Figure 1A). Germline *Sirt5* knockout mice [described in (23)] were used to address whether SIRT5-deficiency affected immune cell development by analyzing thymus and spleen cell contents. The absolute numbers of cells in the thymus and the spleen of SIRT5^+/+^ and SIRT5^−/−^ mice were similar (Figure 1B). When compared to SIRT5^+/+^ mice, SIRT5^−/−^ mice expressed normal proportions and absolute numbers of CD4/CD8 double negative (DN), double positive (DP), and single positive (SP) thymocytes (Figure 1C), such as of DN1-DN4 subpopulations (CD25^+^CD44^+^, CD25^−^CD44^+^, CD25^+^CD44^−^, and CD25^−^CD44^−^). SIRT5^−/−^ mice expressed normal proportions and absolute numbers of splenic CD3^+^ T cells (SP, DN as well as CD4^+^ and CD8^+^ CD44^low^CD62L^high^ naïve and CD44^high^CD62L^low^ memory T cells), B cells (non-IgD^+^/CD23^+^ immature B cells and IgD^+^CD23^+^ mature B cells) and DCs (B220^−^CD11c^+^ conventional DCs and B220^+^CD11c^+^ plasmacytoid DCs) (Figure 1D). Overall, SIRT5 deficiency had no impact on the development of the main T-cell, B-cell and DC populations.

### SIRT5 deficiency does not affect the response of macrophages and splenocytes to immune stimulation

**Figure 2:**
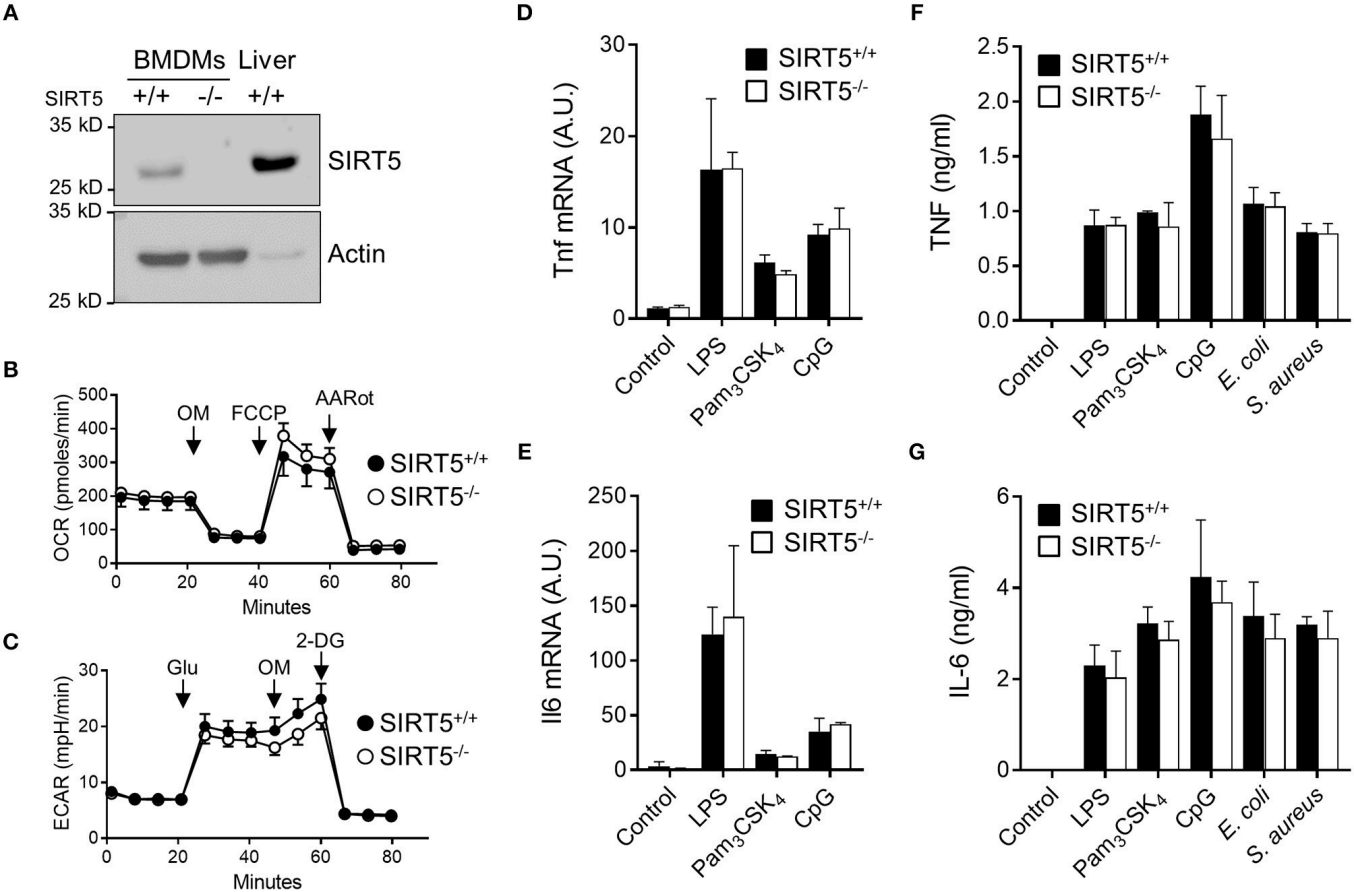
SIRT5 deficiency does not affect TNF and IL-6 production by macrophages exposed to microbial stimuli. **(A)** SIRT5 protein expression in SIRT5^+/+^ and SIRT5^−/−^ BMDMs and SIRT5^+/+^ liver assessed by Western blotting. Full-length blots are presented in Figure S1. **(B,C)** Oxygen consumption rate (OCR, **B**) and extracellular acidification rate (ECAR, **C**) of SIRT5^+/+^ and SIRT5^−/−^ BMDMs. Data are means ± SD from one experiment performed with two mice. **(D–G)** SIRT5^+/+^ and SIRT5^−/−^ BMDMs were exposed to LPS (10 ng/ml), Pam_3_CSK_4_ (10 ng/ml), CpG (2 μg/ml), *E. coli*, and *S. aureus* (10^6^ CFU/ml). Gene expression levels were quantified by RT-PCR, normalized to actin levels, and expressed relative to SIRT5^+/+^ control set at one [**(D)**: 1 h, **(E)**: 4 h]. The concentrations of TNF (4 h) and IL-6 (18 h) in cell culture supernatants were quantified by ELISA **(F,G)**. Data are means ± SD of triplicate samples from one experiment performed with three mice and are representative of two-three experiments **(A, D–G)**. *P* > 0.05 for all conditions.

Macrophages are highly proficient at sensing microbial products through TLRs and play a central role in anti-microbial host defenses by orchestrating innate and adaptive immune responses through the production of cytokines. Bone marrow derived macrophages (BMDMs) expressed SIRT5 protein, albeit less than liver. SIRT5 was undetectable in SIRT5^−/−^ BMDMs (Figure 2A). SIRT5^−/−^ BMDMs showed a slight increased oxygen consumption rate (OCR, readout of mitochondrial activity, Figure 2B) and decreased acidification rate (ECAR, readout of glycolytic activity, Figure 2C).

**Figure 3:**
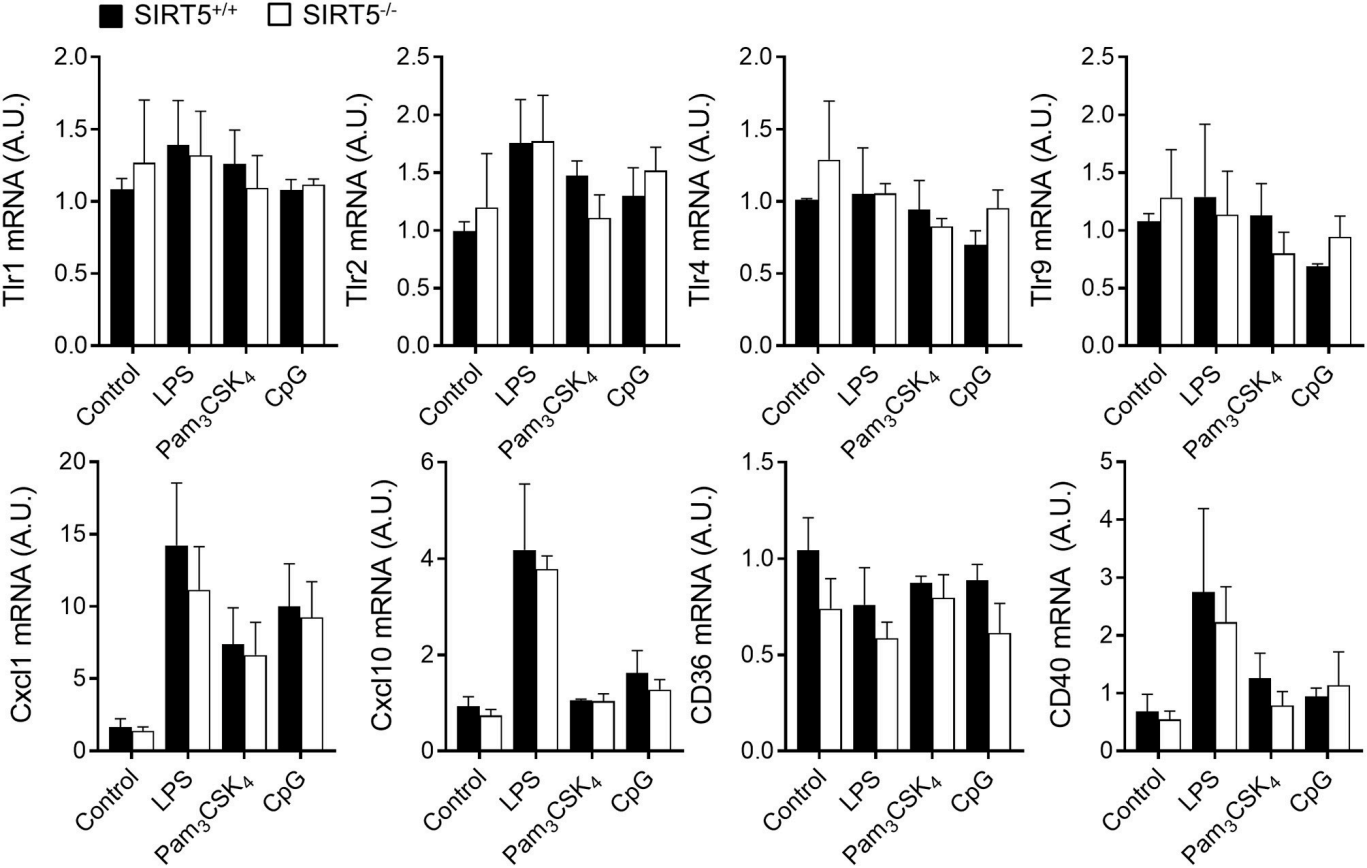
SIRT5 deficiency does not affect macrophage response to microbial stimulation. SIRT5^+/+^ and SIRT5^−/−^ BMDMs were exposed for 1 h to LPS (10 ng/ml), Pam_3_CSK_4_ (10 ng/ml) and CpG (2 μg/ml). Gene expression levels were quantified by RT-PCR, normalized to actin levels, and expressed relative to SIRT5^+/+^ control set at one. Data are means ± SD of triplicate samples from one experiment performed with three mice. *P* > 0.05 for all conditions.

BMDMs were exposed to LPS, Pam_3_CSK_4_, CpG [i.e., TLR4, TLR1/TLR2, and TLR9 agonists (1, 2)] and heat killed bacteria before measuring cytokine response. SIRT5^+/+^ and SIRT5^−/−^ BMDMs up-regulated likewise Tnf and Il6 mRNAs (Figures 2D,E). Moreover, SIRT5^+/+^ and SIRT5^−/−^ BMDMs secreted similar levels of TNF and IL-6 in response to LPS, Pam_3_CSK_4_, CpG, *E. coli* and *S. aureus* (Figures 2F,G). In accordance with these results, SIRT5^+/+^ and SIRT5^−/−^ BMDMs expressed similar levels of Tlr1, Tlr2, Tlr4, and Tlr9 mRNA at baseline and upon exposure to LPS, Pam_3_CSK_4_, and CpG (Figure 3, upper panels). Finally, SIRT5^+/+^ and SIRT5^−/−^ BMDMs cultured with medium, LPS, Pam_3_CSK_4_, and CpG expressed comparable gene expression levels of CXCL1 (KC/GROα) and CXCL10 (IP10) chemokines, CD36 scavenger receptor and CD40 costimulatory molecule (Figure 3, lower panels).

**Figure 4:**
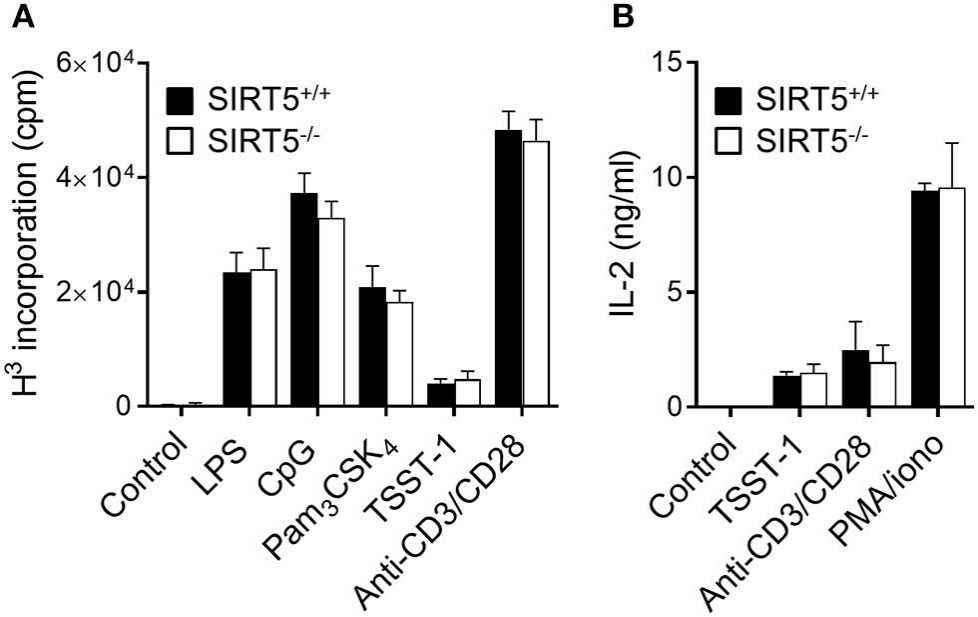
SIRT5 deficiency does not affect proliferation of and IL-2 production by splenocytes. SIRT5^+/+^ and SIRT5^−/−^ splenocytes were incubated for 48 h with LPS (5 μg/ml), CpG (2 μg/ml), Pam_3_CSK_4_ (5 μg/ml), TSST-1 (2 μg/ml), anti-CD3/CD28 antibodies (1μg/ml) and PMA + ionomycin (PMA/iono, 10 ng/ml/100 ng/ml). **(A)** Proliferation was measured by ^3^H-thymidine incorporation. **(B)** IL-2 concentrations in cell culture supernatants were quantified by ELISA. Data are means ± SD of one experiment performed with three mice and are representative of two experiments. *P* > 0.05 for all conditions.

To address further whether SIRT5 deficiency affected the response of immune cells, the proliferation of SIRT5^+/+^ and SIRT5^−/−^ splenocytes exposed to LPS, CpG, Pam_3_CSK_4_, TSST-1, and anti-CD3/CD28 was assessed by ^3^H-thymidine incorporation, (Figure 4A), while the production of IL-2 by SIRT5^+/+^ and SIRT5^−/−^ splenocytes exposed to TSST-1, anti-CD3/CD28 and PMA plus ionomycin was measured by ELISA (Figure 4B). Neither proliferation nor IL-2 production was modified by SIRT5 deficiency. Altogether, the results argued against an important role of SIRT5 in controlling cytokine production by macrophages exposed to TLR ligands and the response of splenocytes to microbial and immune stimuli.

### SIRT5 deficiency does not affect endotoxemia and does not worsen bacterial infections

**Figure 5:**
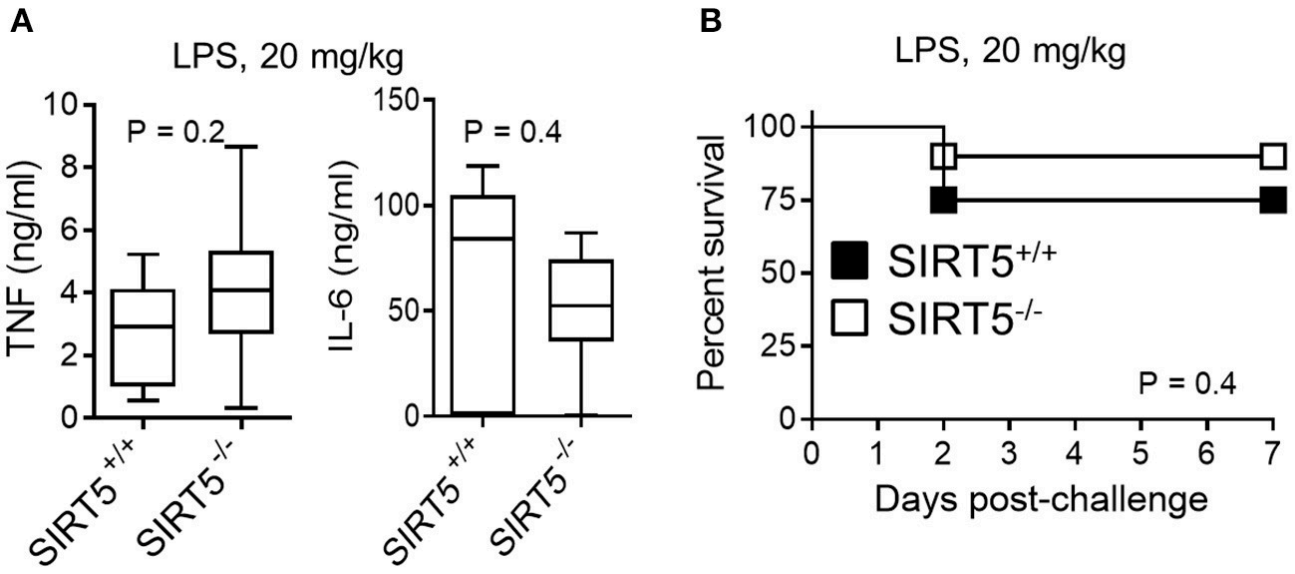
SIRT5 deficiency does not affect endotoxemia. SIRT5^+/+^ and SIRT5^−/−^ female mice were injected i.p. with 20 mg/kg LPS (*n* = 8 and 10 mice per group). **(A)** TNF and IL-6 concentrations in blood collected 1 h (TNF) and 6 h (IL-6) after LPS challenge. *P* = 0.2 and 0.4. **(B)** Survival of mice. *P* = 0.4.

SIRT5^+/+^ and SIRT5^−/−^ mice were subjected to endotoxemia induced by an i.p. challenge with 20 mg/kg LPS. Consistent with the results observed *in vitro*, TNF, and IL-6 concentrations in blood collected 1 h (TNF) and 6 h (IL-6) after LPS challenge were similar in SIRT5^+/+^ and SIRT5^−/−^ mice (*P* = 0.2 and 0.4; Figure 5A). Accordingly, the mortality rates of SIRT5^+/+^ and SIRT5^−/−^ mice were not significantly different (75% vs. 90%; *P* = 0.4; Figure 5B). To mimic clinical situations, we then explored the impact of SIRT5-deficiency on host defenses in models of infections induced by challenging mice with *K. pneumoniae* and *S. pneumoniae* i.n., *E. coli* i.p. and *L. monocytogenes* and *S. aureus* i.v.

**Figure 6:**
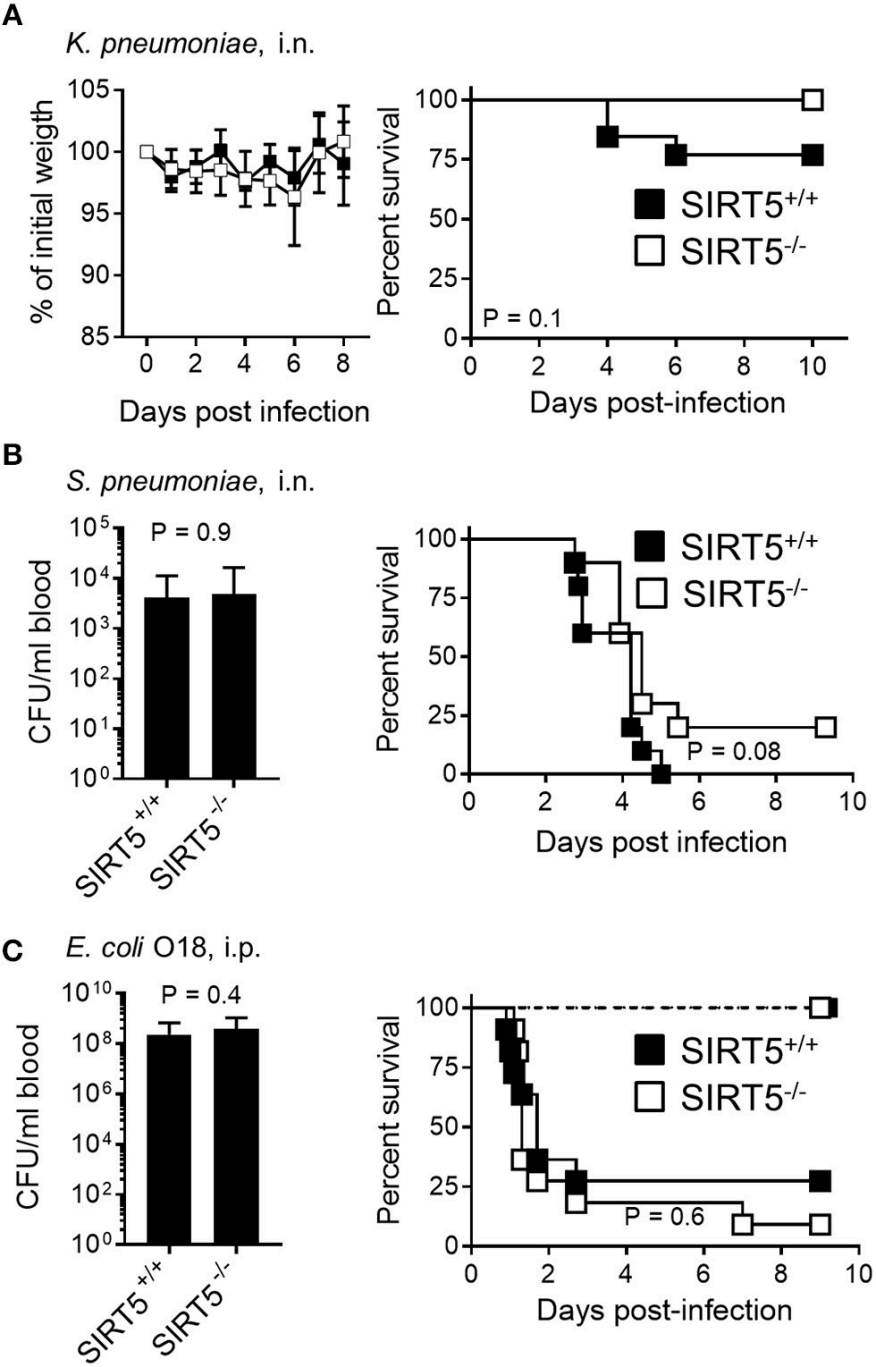
SIRT5 deficiency does not worsen *Klebsiella pneumoniae* and *Streptococcus pneumoniae* pneumonia and *E. coli* peritonitis. SIRT5^+/+^ and SIRT5^−/−^ mice were injected i.n. with 30 CFU *K. pneumoniae* (**A**, *n* = 11 females + 2 males and 7 females + 3 males) or 1.6 × 10^6^ CFU *S. pneumoniae* (**B**, *n* = 10 males per genotype) and i.p. with 4 × 10^2^ CFU *E. coli* [**(C)**, *n* = 7 males per genotype, dashed lines] or 3 × 10^4^ CFU *E. coli* [**C**, *n* = 11 females per genotype, plain lines]. Body weight is expressed in percentage of initial weight (**A**, left panel). Blood was collected 18 h post-infection to quantify bacteria [**(B,C)**, left panels; **(C)**: following infection with 3 × 10^4^ CFU *E. coli. P* = 0.9 and 0.4]. [**(A–C)**, right panels] Survival: *P* > 0.05 for all models.

In a non-severe model of *K. pneumoniae*-induced pneumonia, body weight loss was similar in the SIRT5^+/+^ and SIRT5^−/−^ groups (Figure 6A, left panel). Moreover, mouse survival was not impaired by SIRT5 deficiency (SIRT5^+/+^ vs. SIRT5^−/−^: 77% vs. 100% survival; *P* = 0.1, Figure 6A, right panel). SIRT5 deficiency did not worsen the outcome of mice in a quickly lethal model of *S. pneumoniae*-induced pneumonia (Figure 6B). Two days post-infection, the proportions of bacteremic mice (6/10 vs. 5/10) and blood *S. pneumoniae* loads (SIRT5^+/+^ vs. SIRT5^−/−^: 4.1 ± 2.2 × 10^3^ CFU/ml vs. 4.8 ± 3.6 × 10^3^ CFU/ml; mean ± SEM; *P* = 0.9) were equivalent in the two groups. Accordingly, mortality rate was not significantly different (100 vs. 89% mortality; *P* = 0.08).

**Figure 7:**
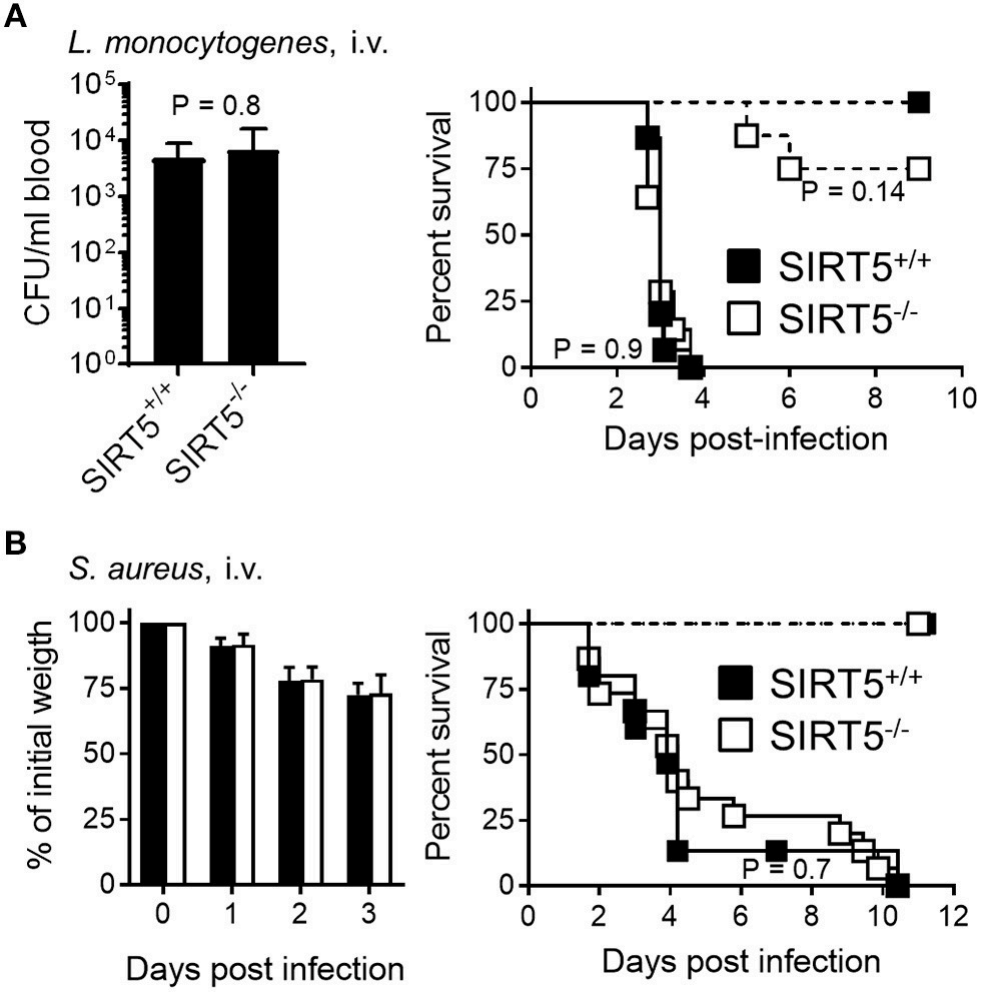
SIRT5 deficiency does not worsen listeriosis and staphylococcal infection. SIRT5^+/+^ and SIRT5^−/−^ mice were injected i.v. with 1.2 × 10^3^ CFU *L. monocytogenes* [**(A)**, *n* = 5 females + 3 males per genotype, dashed lines], 9 × 10^4^ CFU *L. monocytogenes* [**A**, *n* = 15 and 14 females, plain lines], 3 × 10^4^ CFU *S. aureus* (**B**, *n* = 6 females per genotype, dashed lines) or 2 × 10^7^ CFU *S. aureus* (**B**, *n* = 9 females + 6 males per genotype, plain lines, 2 experiments). Blood was collected 48 h post-infection with 9 × 10^4^ CFU *L. monocytogenes* to quantify bacteria (**A**, left panel, *P* = 0.8). Body weight following infection with 2–10 × 10^7^ CFU *S. aureus* is expressed in percentage of initial weight (**B**, left panel). Survival: *P* = 0.14 when comparing SIRT5^+/+^ and SIRT5^−/−^ mice challenged with 1.2 × 10^3^ CFU *L. monocytogenes* and *P* > 0.5 for all other models.

In a model of acute peritonitis induced by *E. coli*, bacterial dissemination into the blood (SIRT5^+/+^ vs. SIRT5^−/−^: 2.3 ± 1.3 × 10^8^ CFU/ml vs. 3.8 ± 2.0 × 10^3^ CFU/ml; mean ± SEM; *P* = 0.4) and mortality rate (73 vs. 91%, *P* = 0.6) were comparable using SIRT5^+/+^ and SIRT5^−/−^ mice (Figure 6C, plain lines). Upon challenge with a low inoculum of *E. coli*, all SIRT5^+/+^ and SIRT5^−/−^ mice survived (Figure 6C, dashed lines). During acute listeriosis, SIRT5^+/+^ and SIRT5^−/−^ mice displayed similar bacteremia (SIRT5^+/+^ vs. SIRT5^−/−^: 4.1 ± 2.2 × 10^3^ CFU/ml vs. 4.8 ± 3.6 × 10^3^ CFU/ml; mean ± SEM, *P* = 0.8) and survival rate (*P* = 0.9) (Figure 7A, plain lines). In a model of sublethal listeriosis, the mortality rate of SIRT5^+/+^ and SIRT5^−/−^ mice was not statistically different (SIRT5^+/+^ vs. SIRT5^−/−^: 100% vs. 75% survival; *P* = 0.14, Figure 7A, dashed lines). In a model of severe, systemic staphylococcal infection (Figure 7B, plain lines), there was no difference in severity score, body weight loss and survival (0 vs. 0%; *P* = 0.7) between SIRT5^+/+^ and SIRT5^−/−^ mice. Analogous to what observed upon challenge with low inocula of *K. pneumoniae, E. coli* and *L. monocytogenes* (Figures 6A,C, 7B), SIRT5^+/+^ and SIRT5^−/−^ mice were similarly resistant to sublethal staphylococcal infection (Figure 7B, dashed lines) suggesting that SIRT5-deficient mice are not particularly susceptible to bacterial infections.

## Discussion

This is the first report of the impact of SIRT5 on antimicrobial host defenses. *In vitro* studies using macrophages and splenocytes and preclinical models of endotoxemia and Gram-positive and Gram-negative bacteria infections suggest that SIRT5 deficiency has no major impact on antibacterial defenses. These observations are particularly relevant in light of the development of pharmacological inhibitors of SIRT5 for clinical applications (42).

SIRT5 deficiency neither affects the development of the major T cells, B cells and DCs subsets in thymus and spleen nor the proliferation and the production of IL-2 by splenocytes. Similar observations were obtained using SIRT2^−/−^ and SIRT3^−/−^ mice (43, 44). In SIRT1-deficient mice, CD4^+^, CD8^+^, and CD4^+^CD8^+^ thymic subpopulations were normal but highly sensitive to DNA-damaging ionizing radiation (45). Circulating T cell, B cell and monocyte counts were normal in 5 months old SIRT7^−/−^ mice that developed inflammatory cardiomyopathy (46). SIRT6-deficent mice developed, after 2 weeks of life, a progeroid syndrome associated with decreased lymphocyte counts in thymus and spleen. However, lymphocyte flaw was not cell-intrinsic but linked to systemic defects (47). Overall sirtuins do not seem to affect the development of adaptive immune cells. Yet, SIRT1 was reported to influence T-helper (Th) 2, Th9, Th17 and T-regulatory (Treg) responses and SIRT3 to sustain the suppressive function of Tregs (48–52). Thus, it would be interesting to define whether SIRT5 shapes T cell responses.

SIRT5 influences diverse metabolic pathways in cardiac and hepatic cells, including urea cycle, amino acid metabolism, the TCA cycle, FAO, glycolysis and oxidative stress response (7–16). SIRT5^−/−^ BMDMs showed a modest increased mitochondrial activity and decreased glycolytic activity, suggesting that SIRT5 may be less influential in macrophages than in heart and liver (8, 15). Correlatively, SIRT5 mRNA and protein were expressed at much lower levels in immune organs [our data and (9)] and in primary macrophages than in liver and heart (5 and 10-fold less SIRT5 mRNA in BMDMs than in liver and heart, respectively).

SIRT5 deficiency had no major impact on LPS-induced cytokine production by macrophages and circulating TNF and IL-6 levels were similar in SIRT5^+/+^ and SIRT5^−/−^ endotoxemic mice. SIRT5-deficient mice under high fat diet, a condition inducing inflammation and oxidative stress, showed normal metabolic parameters and signs of inflammation attested by *Tnf*, *Cd68* (a monocyte/macrophage marker) and *Cd36* (a scavenger receptor) gene expression in the liver (23). Two recent studies analyzed the impact of SIRT5 deficiency on mouse macrophage response to LPS, leading to opposite conclusions. SIRT5^−/−^ peritoneal macrophages produced reduced levels of TNF, IL-6, and MCP-1 (monocyte chemoattractant protein-1/CCL2). SIRT5 competed with SIRT2 to interact with NF-κB p65. Since SIRT2 deacetylates p65 to inhibit its transduction activity, SIRT5 indirectly promoted p65 acetylation and activity (53). In sharp contrast, LPS-stimulated SIRT5^−/−^ BMDMs expressed increased levels of Tnf, Il1b, and Il6 mRNA but not Il10 mRNA. SIRT5 desuccinylated PKM2 (pyruvate kinase M2), promoting tetramer-to-dimer transition and inhibiting pyruvate kinase activity of PKM2. In that study, SIRT5 deficiency protected from DSS-induced colitis. The inconsistency of the impact of SIRT5 on inflammatory responses echoes those reported for SIRT1, SIRT2, SIRT3, and SIRT6 *in vitro* and *in vivo* [discussed in (43, 44)]. Differences in experimental conditions (BMDMs vs. peritoneal macrophages, germline vs. cell-type specific gene knockout, use of si/shRNA and pharmacological modulators of sirtuins) and subtle variations in qualitative and quantitative caloric input and NAD^+^ availability may explain these differences. Additionally, the length of stimulation and the doses of stimulus [10 ng/ml of ultra-pure LPS here vs. 100 ng/ml of crude LPS in (17) and (53)] may have affected the results. It should also be stressed that SIRT5 deficiency was obtained by disruption of exon 4 in the case of the mice used in this study (23), while exons 2–5 were deleted in the SIRT5 knockout mice available from the Jackson Laboratory used in other studies (17, 53). Nonetheless, even in these studies, the background of the animals may have differed substantially considering that commercial knockout mice are of 85% 129 and 15% C57BL/6 backgrounds and that mice were backcrossed 10 times on a BL/6J background in one study (17) while SIRT5^+/+^ and SIRT5^−/−^ littermates were derived from the SIRT5^+/−^ heterozygote mice in the other study (53). Of note, all broad screening proteomic analyses identified metabolic pathways as the most targeted pathways by SIRT5, while pathways commonly associated with immune/inflammatory responses (such as NF-kB, interferon-response, cytokine, cell migration and inflammation pathways) were not evidenced (8, 11, 12, 15, 16).

Endotoxemia reflects pathological situations such as fulminant meningococcemia characterized by high blood loads of endotoxin, but does not reproduce the complex host-pathogen interactions generally taking place during bacterial infections. Therefore, we sought to define the impact of SIRT5 in preclinical models of infections mimicking common clinical situations. SIRT5 deficiency did not sensitize mice to severe *S. pneumoniae* pneumonia, rapidly lethal *E. coli* peritonitis, listeriosis and staphylococcal infection. In the most stringent models, SIRT5 deficiency did not protect from lethal infection, as foreseen if SIRT5 would amplify cytokine response. SIRT5 deficiency also did not render mice particularly susceptible to bacterial infections as suggested by the results obtained using models of sub-lethal/mild infection with *K. pneumoniae, E. coli, L. monocytogenes*, and *S. aureus*. Considering the diversity of the agents (Gram-positive and Gram-negative and intracellular and extracellular bacteria) and of the routes of infection tested (i.n., i.p. and i.v.), these results so far support the assumption that SIRT5 has no dramatic influence on host defenses against bacterial infections and the clinical development of SIRT5 inhibitors for oncologic purposes (18, 22). This contrasts with inhibitors of HDAC1-11 which impaired innate immune defenses against infections in mouse models and have been associated with episodes of severe infection when infused into cancer patients (37, 54–58). Further work will be required to test the efficacy of potential SIRT5 inhibitors (19–21) in models of cancer (18, 22) and of infections and sepsis, then to define whether these inhibitors may predispose to infections in the setting of comorbidities, e.g., in elderly patients and patients with chronic inflammatory disorders like for example colitis and diabetes mellitus.

Overall, SIRT5 does not worsen host defenses to bacterial infections under the conditions tested here. Since sirtuins are linked to metabolism, age-associated dysfunctions and lifespan, it would be of interest to investigate the role of SIRT5 under metabolic stress conditions and in older mice. To conclude, our results support the development of SIRT5 inhibitors for clinical purposes, as they suggest that these drugs would not increase patients' susceptibility to infections.

## Author contributions

TH, EC, CT, AP, and DLR performed *in vitro* experiments. JH participated to flow cytometry analyses. TH, EC, CT, and DLR performed *in vivo* experiments. TR conceived the project, designed the experiments and wrote the paper. All the authors revised the paper.

### Conflict of interest statement

The authors declare that the research was conducted in the absence of any commercial or financial relationships that could be construed as a potential conflict of interest.

## Supplementary material

Figure S1 Full-length Western blots of SIRT5 and actin expression in protein extracts obtained from SIRT5^+/+^ and SIRT5^−/−^ BMDMs and SIRT5^+/+^ liver.

Table S1 Antibodies used for flow cytometry analyses.

Table S2 Oligonucleotides used in RT-PCR analyses.
