# Supplementary figures and images for: Sirtuin 5 Deficiency Does Not Compromise Innate Immune Responses to Bacterial Infections
Source: Front Immunol. 2018 Nov 20;9:2675. doi: 10.3389/fimmu.2018.02675 (PMC6255879; doi:10.3389/fimmu.2018.02675)

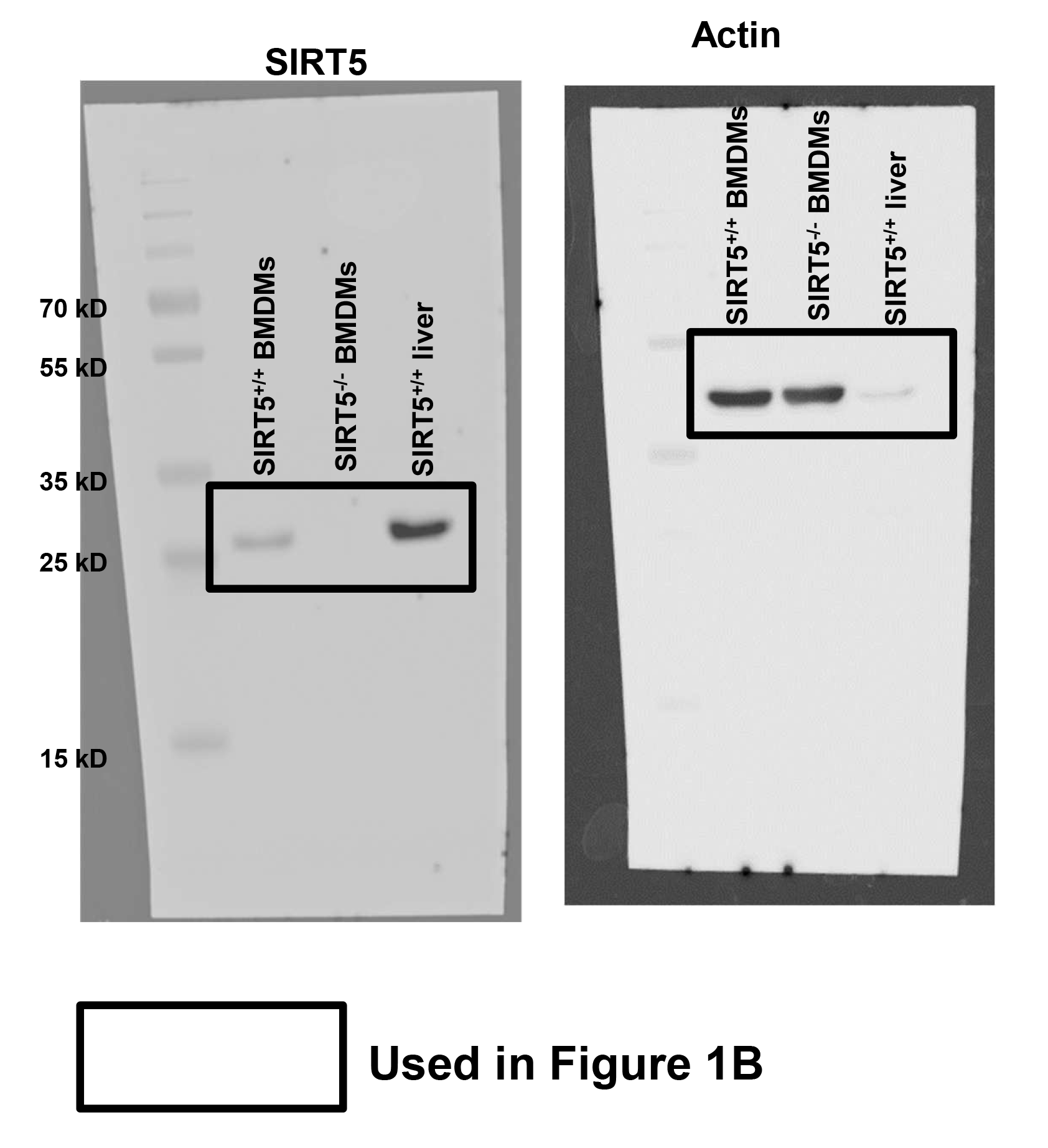

Supplement: Figure S1 — Full-length Western blots of SIRT5 and actin expression in protein extracts obtained from SIRT5+/+ and SIRT5−/− BMDMs and SIRT5+/+ liver. [file Image_1.TIF]
