# Supplementary material for: Sirtuin 5 Deficiency Does Not Compromise Innate Immune Responses to Bacterial Infections
Source: Front Immunol. 2018 Nov 20;9:2675. doi: 10.3389/fimmu.2018.02675 (PMC6255879; doi:10.3389/fimmu.2018.02675)
Supplement: Table S1 — Antibodies used for flow cytometry analyses. [file Table_1.DOCX]

**Supplementary Table S1. Antibodies used for flow cytometry analyses**

| **Target** | **Clone** | **Coupling** | **Company** |
| --- | --- | --- | --- |
| B220 | RA3-6B2 | eFluor® 450 | eBioscience |
| CD3 | 145-2C11 | PE, eFluor® 450 | eBioscience |
| CD4 | RM4-5 | PE, FITC | eBioscience |
| CD8 | 53-6.7 | APC-eFluor® 780, APC-Cy7 | eBioscience |
| CD11b | M1/710 | PE, APC | eBioscience |
| CD11c | HL3 | PE, APC | eBioscience |
| CD14 | Sa2-8 | PE-Cy7 | eBioscience |
| CD23 | B3B4 | PE | eBioscience |
| CD25 | PC61.5 | APC | eBioscience |
| CD44 | IM7 | APC, eFluor® 450 | eBioscience |
| CD62L | MEL-14 | FITC | eBioscience |
| CD93 | AA4.1 | APC | eBioscience |
| IgD | AMS 9.1 | FITC | BD Biosciences |
| MHC-II | 114.15.2 | FITC | eBioscience |

PE: phycoerythrin; FITC: fluorescein isothiocyanate; APC: allophycocyanin.

**Supplementary Table S2. Oligonucleotides used in RT-PCR analyses.**

| **Target** | **Forward primer (5’->3’)** | **Reverse primer (5’->3’)** |
| --- | --- | --- |
| Actin | CGCAAAGACCTGTATGCCAAT | GGGCTGTGATCTCCTTCTGC |
| Cd36 | **TCCCTCACTGGAGGAAACTG** | TGT GAT ATC TGG CCT TGC TG |
| Cd40 | ATGGCCAGTGCTGTGATTTG | GTGGCATTGGGTCTTCTCAAG |
| Cxcl1 | CTTGAAGGTGTTGCCCTCAG | TCTCCGTTACTTGGGGACAC |
| Cxcl10 | GGATGGCTGTCCTAGCTCTGTAC | TGGGCATGGCACATGGT |
| Il6 | CCGGAGAGGAGACTTCACAG | CAGAATTGCCATTGCACAAC |
| Tlr1 | CAGGCGAGCAGAGGCAAT | ATTCCTGAGGTCCCTGCTATTCT |
| Tlr2 | CTTTTCGTTCATCTCTGGAGCAT | GATTTGACGCTTTGTCTGAGGTT |
| Tlr4 | TCAGCAAAGTCCCTGATGACAT | ATGCCATGCCTTGTCTTCAAT |
| Tlr9 | AGGGAGCCTCGGGAGAATC | CCCAGGGCCAGAGTCTCA |
| Tnf | CCAGGCGGTGCCTATGTCT | GGCCATTTGGGAACTTCTCAT |
